# 2833. Multi-drug resistance of *Escherichia coli* from outpatient uncomplicated urinary tract infections in a large U.S. integrated health care organization

**DOI:** 10.1093/ofid/ofad500.2443

**Published:** 2023-11-27

**Authors:** Jennifer H Ku, Katia J Bruxvoort, Suzanne B Salas, Cara D Varley, Joan A Casey, Eva Raphael, Sarah Robinson, Keeve Nachman, Bruno Lewin, Richard Contreras, Rong Wei, Magdalena Pomichowski, Harpreet Takhar, Sara Y Tartof

**Affiliations:** Kaiser Permanente Southern California, Pasadena, California; University of Alabama at Birmingham, Birmingham, Alabama; Kaiser Permanente Southern California, Pasadena, California; Oregon Health and Science University, Portland, Oregon; University of Washington School of Public Health, Seattle, Washington; University of California, San Francisco, San Francisco, California; Sutter Health, Walnut Creek, California; Johns Hopkins Bloomberg School of Public Health, Baltimore, Maryland; Kaiser Permanente Department of Research and Evaluation, Pasadena, CA; Kaiser Permanente Southern California Department of Research & Evaluation, Pasadena, California; Kaiser Permanente Southern California Department of Research & Evaluation, Pasadena, California; Kaiser Permanente Southern California, Pasadena, California; Kaiser Permanente - Southern Calfornia Research And Evaluation - EPI, Pasadena, California; Kaiser Permanente Southern California, Pasadena, California

## Abstract

**Background:**

Urinary tract infections (UTIs) cause significant disease and economic burden. Uncomplicated UTIs (uUTIs) occur in otherwise healthy individuals without underlying structural abnormalities, with uropathogenic *Escherichia coli* (UPEC) accounting for 80% of cases. With recent transitions in healthcare toward virtual visits, data on multi-drug resistance (MDR) (resistant to ≥3 antibiotic classes) by care setting are needed to inform empiric treatment decision-making.

**Methods:**

We evaluated UPEC resistance over time and by care setting (in-person vs. virtual), in adults who received outpatient care for uUTI at Kaiser Permanente Southern California between January 2016 and December 2021.

**Results:**

We included 174,185 individuals who had ≥1 UPEC uUTI (233,974 isolates), who were 92% female, and 46% Hispanic with a mean age 52 years (standard deviation 20). Overall, UPEC MDR decreased during the study period (13 to 12%) (**Figure 1**) both in virtual and in-person settings (p-for trend < 0.001). Resistance to penicillins overall (29%), co-resistance to penicillins and trimethoprim-sulfamethoxazole (TMP-SMX) (12%), and MDR involving penicillins and TMP-SMX plus ≥1 antibiotic class were common (10%) (**Figure 2**). Resistance to 1, 2, 3, and 4 antibiotic classes was found in 19%, 18%, 8%, and 4% of isolates, respectively; 1% were resistant to ≥5 antibiotic classes, and 50% were resistant to none. Similar resistance patterns were observed over time, and by care setting.

**Figure:**
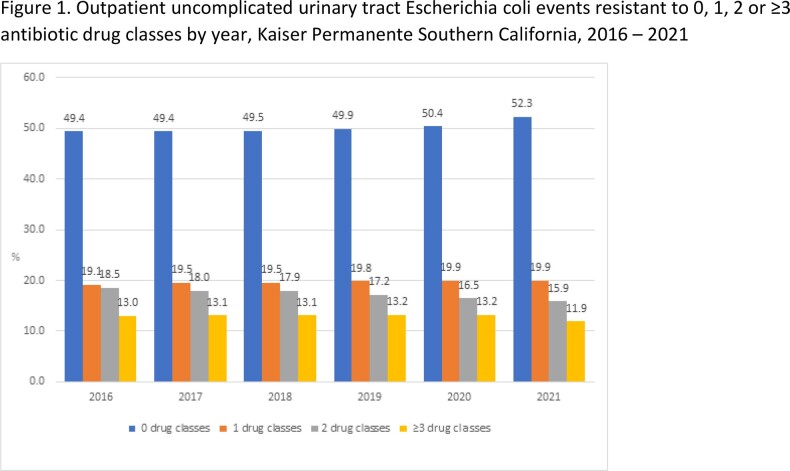

**Figure 2.**
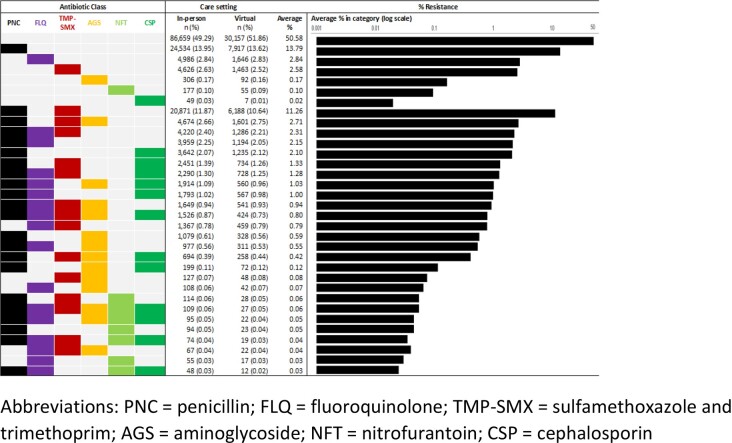
Antibiotic resistance patterns of outpatient uncomplicated Escherichia coli urinary tract events, Kaiser Permanente Southern California, 2016 – 2021. Abbreviations: PNC = penicillin; FLQ = fluoroquinolone; TMP-SMX = sulfamethoxazole and trimethoprim; AGS = aminoglycoside; NFT = nitrofurantoin; CSP = cephalosporin

**Conclusion:**

We observed a slight decrease in both class-specific AMR and MDR of UPEC overall, most commonly involving penicillins and TMP-SMX, and consistent over time and by care setting. Virtual healthcare may expand access to UTI care without increased risk for MDR and the need for setting-specific antibiograms.

**Disclosures:**

**Jennifer H. Ku, PhD MPH**, GlaxoSmithKline: Grant/Research Support|Moderna: Grant/Research Support **Katia J. Bruxvoort, PhD, MPH**, Dynavax: Grant/Research Support|GlaxoSmithKline: Grant/Research Support|Moderna: Grant/Research Support|Pfizer: Grant/Research Support **Sara Y. Tartof, PhD MPH**, Genentech: Grant/Research Support|GSK: Grant/Research Support|Pfizer: Grant/Research Support|SPERO: Grant/Research Support

